# Metagenomic Next-Generation Sequencing of Cerebrospinal Fluid for the Diagnosis of Cerebral Aspergillosis

**DOI:** 10.3389/fmicb.2021.787863

**Published:** 2021-12-24

**Authors:** Xiao-Wei Xing, Su-Fei Yu, Jia-Tang Zhang, Rui-Shu Tan, Yu-Bao Ma, Xia Tian, Rong-Fei Wang, Guo-En Yao, Fang Cui, Qiu-Ping Gui, Sheng-Yuan Yu

**Affiliations:** ^1^Department of Neurology, Hainan Hospital of Chinese PLA General Hospital, Sanya, China; ^2^Department of Clinical Laboratory, Taizhou Hospital of Zhejiang Province Affiliated to Wenzhou Medical University, Taizhou, China; ^3^Department of Neurology, First Medical Center of Chinese PLA General Hospital, Beijing, China; ^4^Chinese PLA Medical School, Beijing, China; ^5^Department of Pathology, First Medical Center of Chinese PLA General Hospital, Beijing, China; ^6^Department of Neurology, Fourth Medical Center of Chinese PLA General Hospital, Beijing, China

**Keywords:** cerebral aspergillosis, metagenomic next-generation sequencing, pathogens, cerebrospinal fluid, diagnosis

## Abstract

**Purpose:** Cerebral aspergillosis (CA) is a rare but often fatal, difficult-to-diagnose, opportunistic infection. The utility of metagenomic next-generation sequencing (mNGS) for diagnosis of CA is unclear. We evaluated the usefulness of mNGS of the cerebrospinal fluid (CSF) for the diagnosis of CA.

**Methods:** This prospective study involved seven consecutive patients with confirmed CA in whom CSF mNGS was performed. Serum (1→3)-β-D-glucan and galactomannan levels were determined, and histopathological examination and mNGS of the CSF were conducted. CSF specimens from three non-infected patients were used as positive controls.

**Results:** mNGS of the CSF was positive in six of the seven confirmed CA cases (85.71% sensitivity). In the cryptococcal meningitis group (control), mNGS of the CSF was positive for *Aspergillus* in two patients (84.62% specificity). The positive likelihood ratio, negative likelihood ratio, and Youden’s index of mNGS for CA in the CSF were 5.565, 0.169, and 0.7, respectively. Among the six mNGS-positive cases, more than two *Aspergillus* species were found in four (4/6, 66.67%). In the positive controls, the addition of one *A. fumigatus* spore yielded a standardised species-specific read number (SDSSRN) of 25.45 by mNGS; the detection rate would be 0.98 if SDSSRN was 2.

**Conclusion:** mNGS facilitates the diagnosis of CA and may reduce the need for cerebral biopsy in patients with suspected CA.

**Trial Registration Number:** Chinese Clinical Trial Registry, ChiCTR1800020442.

## Introduction

Cerebral aspergillosis (CA) is a rare and life-threatening opportunistic infection caused by *Aspergillus* species. This notorious complication of invasive aspergillosis, which accounts for 5–10% of all intracranial fungal pathologies (Ellenbogen et al., 2016), is associated with a >90% mortality rate (Walsh et al., 2008). The major risk factors for invasive aspergillosis include bone marrow transplant (32%), haematological malignancy (29%), solid organ transplant (9%), pulmonary diseases (9%), and acquired immunodeficiency syndrome (8%) (Patterson et al., 2000). CA typically involves haematogenous dissemination from pulmonary lesions, iatrogenic inoculation during surgery or spinal anaesthesia, or direct extension from infections of the ear, orbital, or paranasal sinuses (McCarthy and Walsh, 2017; Winterholler et al., 2017). The gold standard for diagnosing CA is histopathological evidence or a positive culture result for a biopsy or cerebrospinal fluid (CSF) specimen (Walsh et al., 2008). However, these methods are time-consuming, laborious, and have variable sensitivity and specificity (McCarthy and Walsh, 2017). Therefore, confirmation of CA is problematic, and the misdiagnosis rate is high (Wang et al., 2017).

Metagenomic next-generation sequencing (mNGS) enables diagnosis of infectious diseases of the central nervous system (CNS) (Ramachandran and Wilson, 2020) and is increasingly being used in the clinic (Wilson et al., 2014, 2019; Guan et al., 2016; Fan et al., 2018; Wang et al., 2019). mNGS of the CSF can identify pathogens of infectious diseases of the CNS (Xing et al., 2020). However, whether mNGS can detect *Aspergillus*, which is widespread in the environment (Cadena et al., 2016), and assist the diagnosis of CA is unclear. Here, we present seven cases of biopsy-confirmed CA to evaluate the performance of mNGS of CSF for detecting CA.

## Materials and Methods

### Participant Recruitment

We prospectively identified seven consecutive patients with confirmed CA admitted to two teaching hospitals in Beijing, China between November 2016 and September 2019. We recorded the patients’ clinical data, including relevant medical history, physical examination findings, routine blood examinations, CSF parameters, and neuroimaging findings. Diagnosis of CA was confirmed by CSF culture or histopathological evidence. Because of the similar risk factors for CA and cryptococcal meningitis (CM), 13 patients with CM confirmed by CSF India ink smear and/or culture were used as controls.

Sterile CSF was collected from three non-infected patients who required lumbar puncture and divided into three 0.6-mL tubes. Next, different concentrations of *Aspergillus* spores (*A. fumigatus* B5233 wild type) were added to sterile CSF for sensitivity testing of mNGS in spiked specimens and as positive controls. The number of *A. fumigatus* spores in the three 0.6-mL amounts of CSF was 50, 250, and 500.

### Metagenomic Next-Generation Sequencing of Cerebrospinal Fluid

Cerebrospinal fluid specimens were collected from the CA and CM patients in accordance with standard aseptic procedure and subjected to mNGS within 24 h. Next, CSF samples were subjected to bead beating, DNA extraction, DNA library construction, and sequencing (BGISEQ 50 platform; BGI-Tianjin, Tianjin, China). Nucleic acids extracted from blood of healthy volunteers were mixed with sterile water as negative controls. CSF specimens from three non-infected patients were sequenced as positive controls using the MGI DNBSEQ platform (BGI-Tianjin). The procedure has been described in detail elsewhere (Xing et al., 2018, 2019, 2020).

### Interpretation of Metagenomic Next-Generation Sequencing Data

The sequencing data were analysed in terms of species-specific read number (SSRN), genome coverage (%), and depth. *Aspergillus* species with an SSRN ≥ 2 were considered positive. The data interpretation method has been described in detail elsewhere (Xing et al., 2020).

### Statistical Analysis

Continuous data were subjected to non-parametric tests. Quantitative variables are expressed as medians (ranges) and qualitative variables as percentages. Data processing was performed using SPSS software (version 23.0; IBM Corp., Armonk, NY, United States). We performed a linear regression of SSRN and the number of spores that transected the origin. Because the total number of reads obtained from mNGS varied among samples, we first standardised the SSRN to obtain the standardised species-specific read number (SDSSRN) for comparison purposes. The standardised ratio (SR) was calculated as the number of total reads × (1-adaptor ratio)/20,000,000 (Huang et al., 2020). Twenty million was the expected number of total reads after the removal of all adaptors. The SDSSRN was calculated as SSRN/SR, and the spores (*n* = 50, 250, and 500) were mapped to SDSSRNs.

We assessed the probability of *A. fumigatus* detection given an SDSSRN of 2, the former standard for *A. fumigatus* detection. We hypothesised that the probability of mapping a single PMseq read to the *A. fumigatus* genome is a Bernoulli process, as suggested by Ebinger et al., meaning that the read either mapped correctly or incorrectly (Van Borm et al., 2021). Therefore, we expected the detection probability of *A. fumigatus* to follow a binomial mass function with increasing SDSSRN. A binomial distribution function takes into account two parameters: the probability of an event (*p*; a single read successfully mapped to the genome) and the number of trials (*n*; the number of SDSSRNs obtained). For a PMseq read, a DNA sequence of 150 bp on average is produced, and 50 bp at one end is used for genome mapping. Hrant et al. suggested that a 50 bp read obtained by shotgun-sequencing gave a 6% probability of mapping to multiple genomes and 0.52% probability of erroneous mapping (Hovhannisyan et al., 2020), while Hajibabeai et al. suggested that a 109 bp mini-barcode DNA sequence could be used to identify a species with 92% accuracy. We estimated the probability, *p*, that a single read mapped correctly to the *A. fumigatus* genome (indicating *A. fumigatus* positivity) was 0.85. Therefore, we defined the detection probability as [1 – (0.15)^SDSSRN^], where 0.15 is the likelihood that one SDSSRN read was mapped incorrectly.

## Results

### Patients’ Characteristics

Of the seven non-HIV-infected patients identified, three (42.86%) were males. The median age at presentation was 46 (27–80) years. The patients’ characteristics are listed in Table 1. The major symptoms and neurological signs of the seven cases included paralysis of the cranial nerve (6/7, 85.71%), facial pain or headache (4/7, 57.14%), and weakness of the limbs (3/7, 42.86%). Of the seven CA cases, five (5/7, 71.43%) had a history of underlying conditions, including nasal surgery, mastoiditis, diabetes mellitus, excessive alcohol consumption, and septic shock. Of the 13 cases of confirmed CM, 7 (53.85%) had a history of underlying conditions (Table 2).

**TABLE 1:** Clinical features of seven patients with cerebral aspergillosis.

Case no./age (years) /gender	Symptoms and neurological signs	Underlying conditions	MRI findings	Serum BDG (<10 pg/mL)	Serum GM (<0.65 μg/L)
1/40/F	Facial pain, headache, paralysis of cranial nerves (II–VI)	Nasal surgery	Space-occupying lesion of right paranasal sinuses, cavernous sinus, foramina lacerum anterius and temporal lobe, leptomeningeal enhancement	982	ND
2/54/F	Fever (39.5°c) paralysis of cranial nerves (IX, X), limb weakness	DM, mastoiditis	Space-occupying lesion of left tentorium cerebelli, acute cerebral infarction (pons), leptomeningeal enhancement	95.0	0.67
3/46/M	Paralysis of cranial nerves (VI, VII, IX, X), instability of gait, numbness of limbs	Excessive alcohol consumption	Space-occupying lesion of right cerebellum, leptomeningeal enhancement, acute cerebral infarction (right cerebral hemisphere)	108.4	0.937
4/59/F	Headache, ophthalmodynia, proptosis, paralysis of cranial nerves (II–V1)	DM	Space-occupying lesion of left sphenoid sinus and nasopharynx, leptomeningeal and left optic nerve sheath enhancement	27.3	Neg
5/80/F	Headache, paralysis of cranial nerves (II–VI), behavioural change, neck stiffness	Infection of biliary tract and septic shock	Space-occupying lesion of left posterior orbital, cavernous sinus, temporal lobe and anterior skull base, leptomeningeal and optic nerve sheath enhancement	176.3	0.884
6/38/M	Headache, memory impairment, weakness of limbs	Neg	Space-occupying lesion of left frontal and insular lobe, leptomeningeal enhancement	ND	ND
7/27/M	Memory impairment, aphasia, paralysis of cranial nerve (VII), decreased consciousness, weakness of limbs, epilepsy, neck stiffness	Neg	Space-occupying lesion of left frontal lobe, insular lobe and basal ganglia, leptomeningeal enhancement	60.2	<0.25

*BDG, (1→3)-β-D-glucan; DM, diabetes mellitus; F, female; GM, galactomannan; M, male; MRI, magnetic resonance imaging; ND, no data; neg, negative.*

**TABLE 2:** Characteristics of 13 patients with cryptococcal meningitis.

Case no./age (years)/gender	Underlying diseases	India ink staining/CSF culture	mNGS of CSF for *Aspergillus*			
			*Aspergillus* identified	SSRN	Coverage, %	Depth
1/55/M	DM	+/+	−	ND	ND	ND
2/68/F	Polymyalgia rheumatica, IST	+/+	−	ND	ND	ND
3/60/F	−	+/+	−	ND	ND	ND
4/41/M	−	+/+	−	ND	ND	ND
5/66/F	Membranous nephropathy, IST	+/−	−	ND	ND	ND
6/62/F	SLE, IST	+/+	−	ND	ND	ND
7/56/M	DM, CHB	−/+	−	ND	ND	ND
8/15/M	Years of chronic diarrhoea (aetiology unknown)	+/−	−	ND	ND	ND
9/27/M	−	+/+	−	ND	ND	ND
10/54/F	IgA nephropathy, IST	−/+	−	ND	ND	ND
11/30/M	−	+/+	*A. sydowii*	2	0.0003	1
12/41/M	Renal transplantation, IST	+/+	*A. flavus*	5	0.044%	2.8
13/60/M	−	+/+	−	ND	ND	ND

*CHB, chronic hepatitis B; DM, diabetes mellitus; IST, immunosuppressive therapy; SLE, systemic lupus erythematosus; MRI, magnetic resonance imaging; ND, no data.*

**FIGURE 1:**
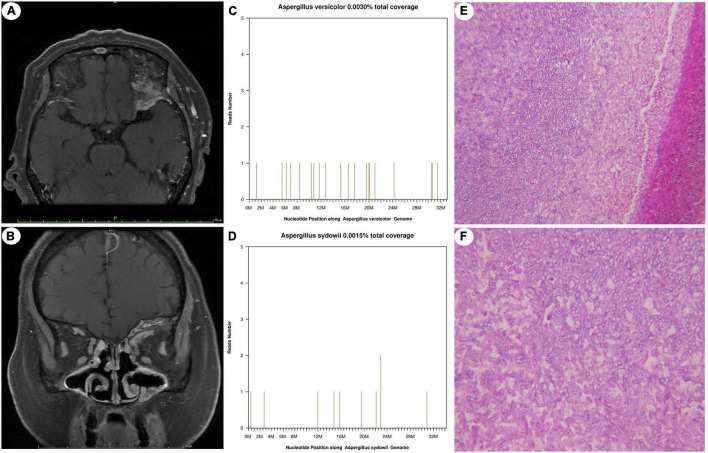
Neuroimaging, mNGS results, and histopathological findings of case 4. T1-weighted MRI with contrast depicting a space-occupying lesion in the left sphenoid sinus and nasopharynx **(A,B)**. The species-specific read numbers of *Aspergillus versicolor* and *Aspergillus sydowii* genomes were 20 and 11, with coverages of 0.0030 and 0.0015%, respectively **(C,D)**. PAS stain demonstrating *Aspergillus* hyphae branching at 45°. Magnification, ×200 **(E)**, ×400 **(F)**.

All of the patients with CA (7/7, 100%) underwent cranial magnetic resonance imaging (MRI) and exhibited space-occupying lesions, including in the paranasal sinuses, cavernous sinus, or skull base in five cases (5/7, 71.43%) and frontal lobe and insular lobe in two cases (2/7, 28.57%) (Figure 1). Leptomeningeal enhancement was noted in seven patients (7/7, 100%). Diffusion-weighted imaging (DWI) hyperintensity was found in two patients (2/7, 28.57%), which was considered as CA-related acute cerebral infarction.

### Laboratory Results

The serum tumour markers of the seven patients with CA were normal. Six patients underwent serum (1→3)-β-D-glucan (BDG) testing, and all were positive (6/6, 100%). Five patients underwent serum galactomannan (GM) testing, and three (3/5, 60%) were positive (Table 1). The CSF laboratory results and histopathological findings of the seven patients are summarised in Table 3. Elevated intracranial pressure (≥200 mmH_2_0) was found in six patients (6/7, 85.71%). The CSF white blood cell count ranged from 0 × 10^6^ to 530 × 10^6/^L (median = 18 × 10^6/^L), the CSF glucose level from 2.4 to 5 mmol/L (median = 3.2 mmol/L), and the CSF protein level from 0.2 to 1.238 g/L (median = 0.878 g/L). All fungal cultures of the CSF were negative (7/7, 100%), and fungal culture of the brain tissue of case 7 was positive for *A. fumigatus*. Twelve of the 13 cases of CM are described in detail elsewhere (Xing et al., 2019). The seven CA patients underwent craniocerebral biopsy, and the histopathological findings showed granulomatous inflammation or inflammatory cell infiltration and *Aspergillus* hyphae (Figure 1). Periodic acid–Schiff (PAS) staining of specimens was positive in six patients (6/7, 85.71%).

**TABLE 3:** Results of CSF analysis and histopathological findings in seven patients with cerebral aspergillosis.

Case no./age (years)/ gender	Routine laboratory CSF evaluations				mNGS of CSF					Histopathological finding/fungal culture
	Pressure (mmH_2_O)	WBC (× 10^6^/L)	Glucose (mmol/L)	Protein (g/L)	Time from onset to CSF collection day	Pathogen identified	SSRN	Coverage, %	Depth	
1/40/F	200	0	3.46	0.2	351	*A. fumigatus*	3	0.0043	1	Granulomatous inflammation; *Aspergillus* hyphae, PAS (+)
						*A. flavus*	2	0.0064	2.4	
						*A. nidulans*	2	0.0038	1	
2/54/F	330	530	5.0	1.114	243	*A. niger*	7	0.0028	1	Granulomatous inflammation; *Aspergillus* hyphae, PAS (+)
3/46/M	280	110	3.2	1.016	228	*A. oryzae*	21	0.0368	1	Granulomatous
						*A. flavus*	8	0.0289	1	inflammation; *Aspergillus* hyphae; PAS (+)
4/59/F	242	5	2.4	0.274	183	*A. versicolor*	20	0.0030	1	Granulomatous
						*A. sydowii*	11	0.0015	1	inflammation; *Aspergillus* hyphae; PAS (+)
5/80/F	75	20	2.6	1.238	272	*A. sydowii*	92	0.0158	1	Inflammatory cell infiltration; *Aspergillus* hyphae; PAS (−)
6/38/M	230	0	3.1	0.572	104	Neg	ND	ND	ND	Inflammatory cell infiltration; *Aspergillus* hyphae; PAS (+)
7/27/M	330	18	3.4	0.878	29	*A. oryzae*	23	0.0279	1	Granulomatous
						*A. flavus*	4	0.0262	1.01	inflammation; *Aspergillus* hyphae; PAS (+); fungal culture of brain tissue (*A. fumigatus)*

*A, Aspergillus; CSF, cerebrospinal fluid; mNGS, metagenomic next-generation sequencing; ND, no data; PAS, periodic acid-Schiff; SSRN, species-specific read number; WBC, white blood cell.*

Of the seven patients with confirmed CA, six exhibited positive mNGS results, for a sensitivity of 85.71%. Species-specific reads mapped onto *A. flavus* (SSRN 2–8) in three cases. Species-specific reads mapped onto *A. sydowii* (SSRN 11–92) and *A. oryzae* (SSRN 21–23) in two cases. Among the six mNGS-positive cases, four (4/6, 66.67%) had more than two *Aspergillus* species. In the six cases with CA, the percentage of SSRNs of *Aspergillus* species (i.e., relative species abundance) was 0.90% (7/774), 31.82% (7/22), 90.91% (30/33), 19.35% (18/93), 73.64% (95/129), and 43.75% (28/64), respectively. Of the 13 patients with CM, *Aspergillus* was found in the CSF of two (Table 2). The specificity, positive likelihood ratio, negative likelihood ratio, and Youden’s index of mNGS for CA in CSF were 84.62%, 5.565, 0.169, and 0.7, respectively.

**FIGURE 2:**
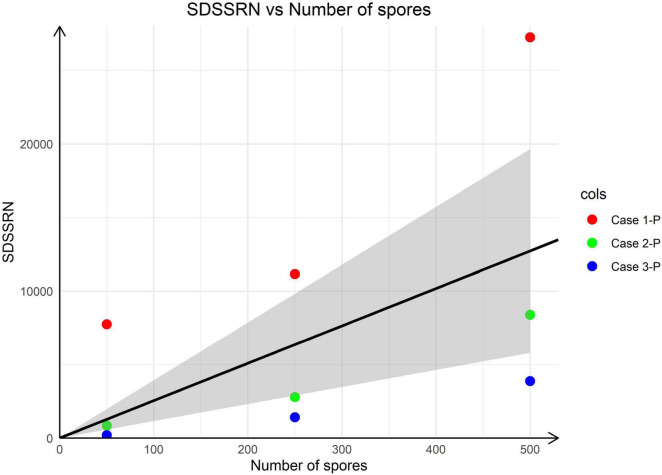
Linear regression of SDSSRN *versus* the number of spores added. The linear regression has the function SDSSRN = 25.45 × (number of spores); the grey area corresponds to the 95% confidence level.

**FIGURE 3:**
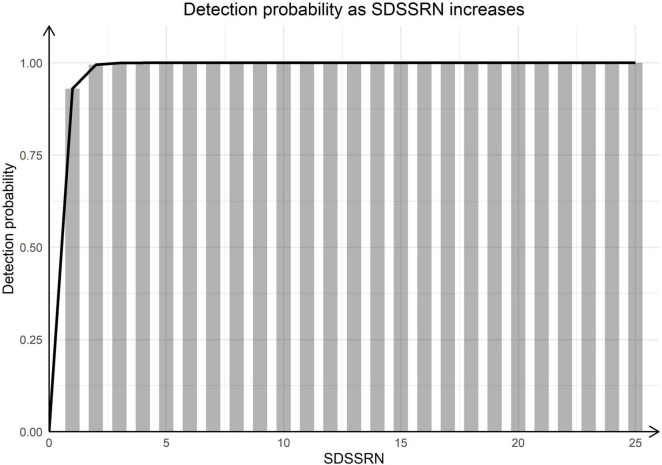
Detection probability with increasing SDSSRN. Bars were calculated as (1–0.15^SDSSRN^).

After dividing SSRN by SR, case 1-P (case1-positive control) had SDSSRNs of 7,754, 11,157, and 27,253 for 50, 250, and 500 added spores, respectively. For case 2-P, the corresponding SDSSRNs were 847, 2,794, and 8,403; and for case 3-P, they were 200, 1,421, and 3,874 (Figure 2). As no SDSSRN should be detected if no spore is added, a linear regression that minimises the total distance of points from the line was drawn through the origin. The linear model had the function SDSSRN = 25.45 × (number of spores), suggesting that the addition of one spore gave an SDSSRN of 25.45 by mNGS. We investigated the *A. fumigatus* detection probability according to SDSSRN. At a *p*-value of 0.85, the detection rate would be 0.98 for an SDSSRN of 2 (Figure 3). Therefore, an SDSSRN of 2 is suitable for *A. fumigatus* detection by mNGS.

## Discussion

Most studies of CA are case reports or case series (Ruhnke et al., 2007; Ellenbogen et al., 2016). In this prospective study, we enrolled seven consecutive CA patients confirmed by biopsy, and 13 confirmed CM, to evaluate the utility of mNGS for the diagnosis of CA. mNGS of CSF contributed to the diagnosis of CA. CA may be caused by several *Aspergillus* species. Moreover, although *Aspergillus* may be present at low abundance, it should not be regarded as background contamination because of the high mortality rate.

Under the updated classification, the genus *Aspergillus* contains 446 species (Houbraken et al., 2020), which are distributed worldwide in various habitats (Samson et al., 2014). The most common pathogenic species are *A. fumigatus, A. flavus, A. niger, A. terreus, A. nidulans*, and *A. sydowii* (Lockhart et al., 2011; Chen et al., 2018). The overall incidence of infections caused by *Aspergillus* is increasing (Chen et al., 2018). Aspergillosis is a life-threatening infection uncommon among the immunocompetent (Bao et al., 2014) but common in the immunocompromised (Patterson et al., 2016).

The clinical presentation of patients with CA is variable and non-specific (Kourkoumpetis et al., 2012), and MRI findings can be helpful for the clinical diagnosis but are nonspecific (Ruhnke et al., 2007). (1→3)-β-D-glucan (BDG), a polysaccharide fungal cell wall component and not specific for *aspergillosis*, can be regarded as a panfungal marker (Lass-Flörl, 2019); the positivity rate was 100% in this study. GM, a carbohydrate component of the cell wall of *Aspergillus* and other fungal species (Ray et al., 2019), is a diagnostic marker for invasive aspergillosis (Patterson et al., 2016); the positivity rate was 60% in this study. Furthermore, GM is not specific to *Aspergillus* and can be positive in infections by other fungi, including *Penicillium marneffei*, *Fusarium*, *Alternaria*, *Histoplasma*, and *Blastomyces* (Barton, 2013).

In view of the non-specificity and low sensitivity of fungal antigen tests, accurate aetiological diagnosis is crucial for the management of CA. *Aspergillus* is rarely detected in cultures of CSF from suspected fungal intracranial infection (Hummel et al., 2006; Ray et al., 2019). The sensitivity and specificity of CSF *Aspergillus* PCR were reported as 75 and 98.3%, respectively, in a case series including five confirmed and seven probable CA cases (Imbert et al., 2017). Nevertheless, *Aspergillus* infection is usually considered only after failure of initial antibiotic treatment for common CNS pathogens (Winterholler et al., 2017). mNGS overcomes these limitations and allows simultaneous and unbiased identification of all microorganisms in human samples (Goldberg et al., 2015).

Our findings show that mNGS enables accurate diagnosis of CA. However, because *Aspergillus* is widely distributed (Samson et al., 2014) and difficult to distinguish from invasive disease, the question arises as to how can we determine that *Aspergillus* detected by mNGS is not a background microorganism. Although CSF is considered aseptic, there may be contaminants, e.g., from skin or laboratory reagents (Ramachandran and Wilson, 2020). Therefore, a strict aseptic and nucleic acid-free standard operating procedure and use of appropriate controls are required for CSF collection and laboratory processing. Also, the clinical significance of detection of *Aspergillus* at low abundance is unclear. Although *Aspergillus* is an opportunistic pathogen, it should not be regarded as background contamination because of the poor outcome of CA. Also, the clinical context is an important matter.

This study involved a relatively large consecutive series of confirmed CA cases. No probable or possible cases were enrolled, enhancing the robustness of the evidence. However, this study had several limitations. First, relatively few patients were enrolled. Second, all *Aspergillus* detected were considered positive mNGS results in the present study. However, whether these opportunistic fungal pathogens are intracranial pathogens is debatable. Third, the utility of an SSRN cut-off value of 2 is unclear. The limited sample size and concentrations of *Aspergillus* spores in the positive controls mean that further study is necessary.

In conclusion, our findings highlight the utility of mNGS of the CSF for non-invasive identification of CA. However, strict aseptic and nucleic acid-free processing and elimination of background contamination are necessary. Pathogen identification should be considered together with the clinical context, such as underlying conditions, symptoms and signs, radiographic evidence, and results of smear, culture, BDG, GM, and other relevant tests.

## Data Availability Statement

The datasets presented in this study can be found in online repositories. The names of the repository/repositories and accession number(s) can be found in the article/supplementary material.

## Ethics Statement

The studies involving human participants were reviewed and approved by the Ethics Committee of the Chinese PLA General Hospital. Written informed consent to participate in this study was provided by the participants’ legal guardian/next of kin.

## Author Contributions

J-TZ designed the study. S-YY organised the experts’ meeting. R-ST, Y-BM, R-FW, G-EY, and FC contributed to the acquisition of clinical data. XT and Q-PG conducted the pathological analysis. S-FY performed the mNGS analysis of positive controls. X-WX conducted analyses and wrote the manuscript. All authors read and approved the final manuscript.

## Conflict of Interest

The authors declare that the research was conducted in the absence of any commercial or financial relationships that could be construed as a potential conflict of interest.

## Publisher’s Note

All claims expressed in this article are solely those of the authors and do not necessarily represent those of their affiliated organizations, or those of the publisher, the editors and the reviewers. Any product that may be evaluated in this article, or claim that may be made by its manufacturer, is not guaranteed or endorsed by the publisher.
